# Will the current National Strategic Plan enable South Africa to end AIDS, Tuberculosis and Sexually Transmitted Infections by 2022?

**DOI:** 10.4102/sajhivmed.v19i1.796

**Published:** 2018-10-04

**Authors:** Kathryn L. Hopkins, Tanya Doherty, Glenda E. Gray

**Affiliations:** 1 Perinatal HIV Research Unit, Faculty of Health Sciences, University of the Witwatersrand, South Africa; 2 School of Public Health, Faculty of Health Sciences, University of the Witwatersrand, South Africa; 3 Health Systems Research Unit, South African Medical Research Council, South Africa; 4 School of Public Health, University of the Western Cape, South Africa; 5 Office of the President, South African Medical Research Council, Cape Town, South Africa

## Abstract

**Background:**

In May 2017, the South African National AIDS Council released the fourth National Strategic Plan (NSP) for HIV, tuberculosis and sexually transmitted infections. This five-year plan (2017–2022), which aims to track the progress towards transitioning these epidemics to no longer being public health threats by the year 2030, is built on the successes and barriers of the previous NSP (2012–2016). However, the NSP does not address some critical components, which may contribute to a future failure in achieving its hefty goals.

**Objectives:**

This article outlines the gaps within the new NSP, as well as highlighting aspects requiring careful focus, which are critical to address in order for South Africa to make progress towards the set targets.

**Method:**

This commentary included an in depth review of the NSP, other South African National Strategic Plans and documents, and scientific literature.

**Results:**

The NSP does not address gaps in funding, oversights in prevention and treatment strategies, human resource shortages and lacking health system requirements.

**Conclusion:**

To realistically achieve the NSP targets and goals, a robust, client-centred strategy addressing the NSP gaps needs to be implemented. The strategy must be cost-effective; provide active linkage to care; and address health system weaknesses that inhibit its successful implementation, including human resources, service delivery and supply chain management, accountability and monitoring and evaluation (M&E).

## Fourth National Strategic Plan

In May 2017, the South African National AIDS Council released the fourth National Strategic Plan (NSP) for HIV, tuberculosis (TB) and sexually transmitted infections (STIs). This five-year plan (2017–2022), which aims to track the progress towards transitioning these epidemics to no longer being public health threats by the year 2030, is built on the successes and barriers of the previous NSP (2012–2016).^1,2^ However, the NSP does not address some critical components, which may contribute to a future failure in achieving its hefty goals. This article outlines the gaps within the new NSP (Table 1), as well as highlighting aspects requiring careful focus, in order for South Africa to make progress towards the set targets.

**TABLE 1:** Summary of gaps within the National Strategic Plan.

NSP topic and proposal	Identified gaps and issues
**Prevention and treatment**	
Reduce STIs: *Treponema pallidum, Neisseria gonorrhoeae, Chlamydia trachomatis.* Eliminate congenital syphilis. 90% national coverage of HPV immunisation programme. Increase detection/treatment of highly prevalent and asymptomatic STIs.	Key prevention interventions still remain at low coverage. PrEP is not available nationwide. Partner-notification strategies are lacking. No baseline STI service coverage data. Low cervical cancer screening coverage. HPV screening is not policy.
**Testing modalities**	
PICT for ANC settings. Increase laboratory support and POC testing, specifically for common STIs.	PICT should be standardised across other services within health facilities. within health facilities. Gold standard POC testing kits for STIs are not yet specified.
**Linkage to care**	
Improve linkage to care to expedite ART initiation Focus on same-day initiation. Use PLWH to help link diagnosed patients to care	Standard of care is passive linkage to care. Active linkage to care strategies is not defined.
**Service delivery and supply chain management**	
Strengthen and enhance efforts to reduce stock-outs Create a national supply chain task force. Support a centralised medicine distribution system.	A national supply chain strategic plan is needed. Current deficiencies in provincial supply chains and emergency management response. Root causes of poor supply chain management have not been pinpointed.
**Human resources**	
Calls for growth and diversification of trained healthcare professionals.	Does not address reasons for current shortage in human resources: Personnel hiring limitations because of fiscal constraints. National policy of CHWs not yet finalised. Increased health personnel costs resulting from 2015 Wage Agreement.
**Health information systems**	
Calls for data-driven action.	Lacks a strong M&E framework No M&E indicators for linkage to prevention, care and treatment, or retention in care. No guidance on how patients should be tracked through continuum of care. No standardised national data capturing system.
**Financing**	
Total cost of NSP over 5 years is R207 billion.	Does not account for the projected gap of a few billion rand, annually: Domestic fiscal spending ceiling. Unreliable foreign aid contributions. Cost-saving strategies need to be implemented.

NSP, National Strategic Plan; STIs, sexually transmitted infections; HPV, human papilloma virus; PrEP, pre-exposure prophylaxis; PICT, provider-initiated counselling and testing; ANC, Antenatal Care; CHW, Community Health Worker; POC, point-of-care; PLWH, people living with HIV and/or AIDS; M&E, monitoring and evaluation.

## Today’s epidemics: Where are we now?

South Africa’s successes since the previous NSP include a 34% and 15% decrease in new HIV infections and TB incidence, respectively.^1^ By December 2016, South Africa had 3.9 million people initiated on antiretroviral treatment (ART) – the largest HIV treatment programme globally.^1,3^ Increased coverage of ART and combination prevention strategies, such as nationwide campaigns to scale up voluntary medical male circumcision (VMMC) (2.4 million procedures conducted) and the distribution of male and female condoms, has led to a decline in new HIV infections.^1,4^ Mathematical modelling on the new Universal Test and Treat (UTT) guidelines, which allow for initiation of treatment irrespective of CD4+ count, has shown that UTT could lead to substantial reductions in HIV incidence, potentially eliminating it as a public health problem over a period of 15–20 years.^5^ The decreased TB incidence could be attributed to the national treatment success rate rising to 83% by 2016 – with some districts exceeding the End TB Strategy target of 90%.^6^ New diagnostic tests, including the rapid GeneXpert technology, have led to more efficient diagnosis and initiation of treatment of new smear-positive and drug-resistant TB cases, which are responsible for the majority of TB transmissions.^1,6,7^ This rapid diagnosis and increased treatment success rate has contributed to the decline in numbers of new TB cases nationwide.^6^

Although TB incidence and number of new HIV infections have declined, they have fallen short of the 50% reduction target.^1,2^ The number of new HIV, TB and STI infections remains unacceptably high (270 000 new cases of HIV in 2016; 450 000 new cases of TB in 2015; and 1.4 million STIs treated annually in public health facilities).^1^ South Africa remains the country with the largest number of HIV/AIDS infections globally, with 18% of adults aged 15–49 years HIV-infected.^1,8,9^ While HIV incidence is decreasing, the prevalence is increasing because of a recovering life expectancy (from 58.3 to 62.4 years between 2011 and 2015), as 50% of people living with HIV/AIDS (PLWH) are on life-saving therapy.^1^ TB is currently the leading cause of death within South Africa, with 63% of TB incidence occurring in PLWH, the largest number of HIV-associated TB cases globally.^1,10^ Eclipsing the (modest) decline in TB incidence since 2012 is an increased prevalence of multidrug-resistant TB – the number of cases have doubled between 2007 and 2012.^1^ Less data are available on South Africa’s STI epidemic; however, it remains a serious public health problem and risk factor for HIV infection.

The earlier age of sexual debut and low condom use may have reduced the success of prevention efforts. The 2016 Demographic and Health Survey found that only 58% of women and 65% of men with multiple sexual partners within the past year reported using a condom during their last sexual intercourse. The number of sexually active men aged 15–49 years with multiple sexual partners has increased, with the mean number of lifetime partners reported as 14.7. South Africa has a high prevalence of gender-based violence, with one in five partnered women and 40% of divorced or separated women having experienced physical violence.^11^ HIV transmission dynamics in South Africa include cyclical intergenerational sex.^12^ Analysis from the South African National HIV Surveys 2002–2012 show younger women (aged 15–24 years) with age-disparate partners (5 + years senior) have an increased risk of acquiring HIV, with the odds of HIV infection increasing for each year increase in the male partner’s age.^13^ These infected young women then have same-age relationships with and infect male partners, who in turn infect younger women.^14^ In 2014, South Africa launched a VMMC campaign targeting the circumcision of 4.3 million men by 2016.^15^ Uptake fell short, especially in traditionally non-circumcising regions,^16^ because of pain, the six-week healing period requiring abstinence and perceptions regarding traditional circumcision versus medical circumcision.^17,18^

## Setting South Africa up to fail?

The NSP 2017–2022 has put forth eight goals, which include breaking the cycle of transmission; reaching the 90-90-90 targets in every district; having targeted interventions reaching all key and vulnerable populations; utilising a multidepartment and multisectoral approach; implementing equal treatment and social justice; promoting leadership and shared accountability; mobilising resources and maximising efficiencies for a sustainable response; and taking data-driven action.^1^ We argue that there are major gaps in the prevention and treatment goals, as well as glaring health system constraints to achieving these goals, that deserve closer scrutiny. Human immunodeficiency virus lobbyists and healthcare organisations have challenged the government publicly because of its failure in considering these aforementioned gaps.^19^

### Gaps in prevention and treatment goals

The NSP focuses on five STIs and holds the following primary objectives: the reduction of *Treponema pallidum, Neisseria gonorrhoeae* and *Chlamydia trachomatis*; elimination of congenital syphilis; and 90% national coverage of the human papilloma virus (HPV) immunisation programme (of Grade 4 learners in South Africa).^1^ The South Africa HIV Investment Case states that the most cost-effective intervention options, which are also applicable to combating the spread of STIs, are those that prevent new infections and thus translate into treatment savings costs (e.g. condom availability and VMMC).^20^ However, these key prevention interventions still remain at low coverage; with expansion efforts – the scaling up of these prevention interventions – only having recently begun.^20^ Furthermore, pre-exposure prophylaxis is not yet available nationally but is only provided through demonstration projects targeting young women, sex workers and men who have sex with men.^21,22^ Details of proposed implementation for partner-notification strategies for HIV and TB infections and STIs are lacking.^1^ HIV and STI partner notification in South Africa largely depends on the patients diagnosed with infections notifying their own sex partners, as opposed to provider-assisted partner notification.^23,24^ Studies in South Africa show that up to 50% of persons diagnosed with STIs have no intention of notifying their partner.^25^

An objective of the NSP is to increase the detection and treatment of highly prevalent and largely asymptomatic STIs such as chlamydia and HPV (prevalence of 17% – 42% and 71% in young women, respectively)^26^ by 50%;^1^ however, there is no fleshed-out strategy to achieve this. Additionally, no baseline STI service coverage data currently exist in South Africa.^1^ Women need increased access to screening for HPV and cervical cancer in order to obtain referrals to care and treatment, if required. While not policy, given HPV’s association with cervical cancer, screening women for HPV would help identify those at risk. These women could then be screened for cervical cancer in government facilities more frequently than current guidelines allow – up to three times in a lifetime (if HIV-uninfected).^27^ Routine facility data from the District Health Information System show that cervical screening coverage among women aged 30 + years was 56%.^28^ This coverage rate may be an overestimate, as smears done more than once in 10 years in the same women, smears done for women under the age of 30 years and repeat smears done because of poor sample quality could inflate the numerator. Even once diagnosed, passive referral is widely implemented in South Africa, across all health domains.

A wider range of testing modalities must be used to reach NSP and global targets for increased disease detection for HIV, TB and STIs.^20^ The South Africa HIV Investment Case communicated that various sub-options may be developed to help reach targets – provider-initiated counselling and testing (PICT) in key populations (e.g. young women) with cost-effective point-of-care (POC) rapid screening technology could be proposed.^20^

The NSP encourages PICT in the antenatal care setting to ensure 100% birth testing of newborns exposed to HIV, as well as PICT of mothers and testing of all children up to 18 months to identify those who have acquired HIV through breastfeeding.^1^ However, PICT is not standardised across other services within health facilities^29^ where the standard of care is usually client-initiated counselling and testing. The World Health Organization (WHO) recommends PICT as an effective public health intervention to increase access to counselling and reduce missed opportunities for testing.^30^

In resource-poor settings, POC technology can offer inexpensive, large-scale screening programmes for HIV, TB, STIs and other emerging infections by expanding critical laboratory testing to rural areas and providing real-time results.^31,32^ For some POC testing, the unit cost per test may be larger through the loss of bulk testing in laboratories, but it offers potentially substantial savings through enabling rapid delivery of results and reduction of facility and personnel costs.^33^ The NSP does call for increased laboratory support and use of POC testing methods, specifically for common STIs,^1^ but it does not explicitly state which testing kits should be used as the gold standard. It is not yet standard of care. For example, with public sector pricing in South Africa, the rapid GeneXpert technology for TB screening costs $14.93 per sample, compared to $16.88 per sample using conventional automated liquid culture-based methods in the laboratory. Additional benefits of the GeneXpert are reduced staff time needed for testing and the potential to increase volume of testing.^34^

The NSP calls for improvement in linkage to care to ensure expedited initiation of ART for all people diagnosed with HIV, as soon as they are ready for treatment and preferably same day. However, it doesn’t explicitly offer innovative ways to link people to services, outside of using ‘PLWH to enhance linkage to care’.^1^ Additional guidance is needed. A systematic review and meta-analysis of community and facility-based HIV testing and linkage to care gaps in sub-Saharan Africa reported that facilitated linkage (e.g. clinic staff actively scheduling a referral visit or accompanying the patient to that visit) achieved both higher linkage to care and ART initiation than passive referral (e.g. providing clients with phone numbers and referral information).^35^

### Lacking health system requirements

Achieving the NSP goals requires attention to critical health system requirements. We have used the WHO health system building blocks^36^ to frame the discussion around these requirements, which include service delivery and supply chain management; health workforce; health information systems; financing and leadership or governance.

## Service delivery and supply chain management

Delivering the services outlined in the NSP requires a well-functioning supply chain. In the Stop Stockouts Project (SSP) Stock Outs National Survey (June 2016), implemented in the last quarter of 2015, health facilities nationwide reported the following when queried about the last three months prior to contact: one in five facilities reported a stock-out of adult antiretrovirals (ARVs) (472/1875), and 70% of facilities reported the stock-out lasting longer than one month (529/760).^37^ Nearly one in five stock-outs of ARVs or TB medicines resulted in the patient leaving the health facility without medication.^37^ The three provinces with the highest frequency of stock-outs (Mpumalanga, Gauteng and the Free State) reported stock-outs lasting for longer than one month, depicting deficiencies in the provincial supply chain and a failure in emergency response mechanisms.^37^ Stock-outs included few fixed-dose combination tablets and consisted of mainly single medications.^38^

In the aftermath of the SSP National Survey, the National Department of Health (NDoH) created a national supply chain task force to initiate supply chain reforms. In partnership, the SSP was granted R300 million to develop a national electronic medicine stock management system.^39^ The NSP declares that steps will be taken to strengthen and enhance efforts to reduce stock-outs, such as an early warning system to identify impending shortages and a distribution system for delivery of medicines for chronic diseases to health facilities – the Central Chronic Medicine Dispensing and Distribution Programme.^1^ While these efforts should have a positive impact on reducing stock-outs of essential medications in South Africa, additional strategies are required to address the root causes of stock-outs in the supply chain, improve and implement monitoring tools to track existing and potential issues in essential medication supply, and work closely with stakeholders, including the NDoH, to develop and implement a national supply chain strategic plan.^37,39^

## Health workforce

The NSP requires a substantial growth and diversification in human resources and commits to invest greater resources towards the training and mobilisation of these personnel. However, it avoids addressing any reasons for the current shortages of healthcare workers – both political and budgetary in nature – which may serve as barriers to its goals.^19^ South Africa has significantly lower numbers of doctors than most income-comparable (upper middle income) countries.^40^ The severe shortage in human resources is compounded by personnel hiring limitations put in place across the NDoH because of fiscal constraints in the 2016 budget;^20^ simply put, there is not enough manpower to implement the NSP.^19,20^

The NSP implementation period coincides with a major health reform process in South Africa, namely the implementation of the National Health Insurance Policy. This policy, which includes contracting with private general practitioners and public clinics, has the potential to assist with skilled staffing shortages provided favourable contracting conditions.^41^ Substantial cost-savings have been reached through shifts towards nurse-based care and treatment and further task-shifting of POC testing to non-healthcare workers (lay counsellors).^20,42,43^

A further cadre requiring greater legislative security are Community Health Workers (CHWs), who are being scaled up as part of ward-based outreach teams. These workers are responsible for contact tracing and counselling, but the national policy on CHWs is still not finalised and there are large provincial differences in contracting methods and salary. The current ratio of CHWs is far too small per population for impact, given their large and increasing scope of work.^44^

South Africa’s health sector budget also needs to manage the increase in health personnel costs resulting from the 2015 wage agreement, which is expected to result in a 7.4% annual growth in wages across government.^45^

## Health information systems

Data-driven action relies on monitoring and evaluation (M&E) of programmatic service delivery to identify service utilisation gaps and outcomes. However, there is little to no mention of NSP M&E indicators tracking linkage to prevention, care and treatment services or retention in care. Within the NSP’s M&E framework indicators, there is no definition for ‘lost to follow-up’ or guidance on how patients should be tracked and data collected throughout the continuum of care.

Where resources are available, an electronic data capturing system can promote the timely submission and analysis of clinical data required for health service delivery improvements, as has been implemented in the Western Cape. The province introduced an award-winning offline electronic version of the typically used paper register system in many healthcare facilities: the Primary Health Care Information System (PHCIS). The PHCIS is now the most widely used patient-based system in South Africa and on the continent, with the patient master index having records for over 80% of patients in the Western Cape. Patient information can be shared across clinics irrespective of location.^46,47^ It incorporates a three-tiered monitoring approach, allowing the Ministries of Health to strategically implement one of the tiers in each facility offering ART services. Each tier has the ability to produce the same nationally required monthly enrolment and quarterly cohort reports to assist in aggregating all tier outputs into a single database at any level of the health system. Implementing such a system at country level for pre-antiretroviral wellness, ART, TB and so on can be an efficient approach to ensuring system-wide synchronisation and accurate M&E of services, including long term retention in care.^47^

## Financing

In 2016, the health sector’s share of the national budget sat at only 13.1% (R159.4 billion).^20^ Of that share, the HIV and AIDS programme allocations made by the NDoH were R17.5 billion.^20^ Health spending is highly dependent on economic growth, with which South Africa is struggling. The current national gross domestic product (GDP) and existing ratios of expenditure estimate that a 1% growth in GDP equates to a R1.5 billion real increase in public health spending annually. Unfortunately, actual health budget increases are less, as a result of the revenue growth being used to decrease national deficits and pay higher interest rates.^20^ It is expected there will only be an annual growth of between 0.4% and 1.3% over the medium term (covering the three financial years from 2016 and 2017 through 2018 and 2019), equating to R913 million per annum for the HIV and AIDS programme.^20^

The total cost of implementing the proposed NSP over the five-year period is a staggering R207 billion, which constitutes a major funding gap. Annual cost estimates rise from R35.1 billion to R45.7 billion, with two goals (prevention and treatment) accounting for 86.5% of the projected budget.^1^

NSP costing does not fully account for the lack of available government funding and foreign development assistance. While South Africa has increased national spending, the government has also declared adherence to a fiscal spending ceiling, as previously budgeted.^20^ Mobilisation of additional domestic resources is limited by the country’s low economic growth, the weakened rand, and high levels of government debt and low credit ratings, which have both increased the cost of national borrowing.^1,20^ Foreign development assistance is uncertain at best. Historically, South Africa received significant contributions from two main funders – the President’s Emergency Plan for AIDS Relief (PEPFAR) and the Global Fund to Fight AIDS, Tuberculosis and Malaria, both of which are now simultaneously decreasing monetary support.^1,20^ The bilateral Partnership Framework Implementation Plan with PEPFAR projected a funding decline from $450 million to $250 million over five years.

The broad NSP has vague strategies (such as the language surrounding the need for provision of condoms within schools and the decriminalisation of sex work while lacking concrete plans on how to do so) with unknown associated costs.^48,49^ The South African HIV and TB Investment Case conducted by the NDoH depicts the gap of a few billion rand, annually, between required funding and projected available amounts.^50^ Cost-savings strategies should focus on improving return on investments by refining the efficiency of current services to deliver high-value, high-impact interventions.^1,51^

## Summary

To realistically achieve the NSP targets and goals, a robust, client-centred strategy addressing the NSP gaps needs to be implemented. The strategy must be cost-effective; provide active linkage to care; and address health system weaknesses that inhibit its successful implementation, including human resources, service delivery and supply chain management, accountability and M&E.
